# Global burden of disease attributable to high fasting plasma glucose from 1990 to 2021: a spatiotemporal analysis of global burden of disease 2021

**DOI:** 10.3389/fcvm.2026.1679255

**Published:** 2026-02-17

**Authors:** Nan Wang, YuYing Wang, QianQian Lv, ZhaoXia Zhang, WenJuan Yang, Jie Wang, Jie Zhang, Nuerbiya Asihaer, Ya Shi, Lu Zhang, JingYi Gao, Ying Xing

**Affiliations:** Department of Endocrinology, Xi'an Daxing Hospital Affiliated to Yan'an University, Xi‘an, China

**Keywords:** age-standardized DALY rates, age-standardized mortality rates, disease burden, future trends, globalburden of disease, high fasting plasma glucose

## Abstract

**Objective:**

This study comprehensively assessed the disease burden attributable to high fasting plasma glucose (HFPG) from 1990 to 2021 and projected its future trends over the next 30 years.

**Research design and methods:**

The analysis was based on data from the Global Burden of Disease (GBD) 2021 study, including mortality, disability-adjusted life years (DALYs), age-standardized mortality rates (ASMR), and age-standardized DALY rates (ASDR). The DisMod-MR 2.1 model was used to estimate the disease burden attributable to HFPG from 1990 to 2021. In addition, an age-period-cohort model was applied to project the disease burden for 2051.

**Results:**

Diabetes mellitus (DM) remained the leading cause of HFPG-attributable burden, with an ASDR of 916.14 (95% UI: 776.14–1,096.55) and an ASMR of 19.61 (95% UI: 18.08–20.82) per 100,000 population. Ischemic heart disease (IHD) and stroke also contributed substantially to global mortality and morbidity. North Africa and the Middle East, along with Oceania, reported the highest regional burdens. China recorded the highest number of deaths (956,264.44) and DALYs (27,655,530.55). Males and individuals aged ≥75 years experienced disproportionately higher burdens. Projections indicated a global decline in ASMR but a continued increase in ASDR, with persistent sex disparities.

**Conclusions:**

DM, IHD, and stroke were the primary contributors to the HFPG-attributable disease burden. Although ASMR is projected to decline, the continued rise in ASDR underscores the need for strengthened prevention strategies and health system responses.

## Introduction

The clinical spectrum of hyperglycemia includes impaired fasting glucose (≥5.6–<7.0 mmol/L) and diabetes (≥7.0 mmol/L). For this study, we adopt the GBD 2021 framework, which defines the exposure of interest as fasting plasma glucose exceeding the theoretical minimum risk exposure level (TMREL) of 4.9–5.3 mmol/L (1–3). HFPG is a well-established driver of several non-communicable diseases, particularly diabetes mellitus (DM), ischemic heart disease (IHD), stroke, and chronic kidney disease (CKD) (4, 5). Mechanistically, chronic hyperglycemia induces systemic damage through pathways such as mitochondrial dysfunction, oxidative stress, and accumulation of advanced glycation end products, which are implicated in diabetic nephropathy, retinopathy, and cardiovascular complications (6).

According to the GBD 2019 study, HFPG ranked as the sixth leading global risk factor for mortality, accounting for approximately 6.5 million deaths and nearly 200 million disability-adjusted life years (DALYs) in 2019—almost double the figures reported in 1990 (2). Regional disparities were pronounced: in the Americas, HFPG-related DALYs increased by 27.4% from 1990 to 2019, with Central America and the Caribbean recording the highest age-standardized ASDR at 326.4 per 100,000 population (7). In China, cardiovascular deaths attributable to HFPG rose sharply in 2018, particularly among adults aged ≥80 years (8). Moreover, GBD 2021 data indicated that between 1990 and 2021, pancreatic cancer deaths attributable to HFPG in China increased by 232%, and related DALYs by 195.4%, underscoring its growing impact on oncologic burden (9).

Despite its escalating health burden, HFPG has received limited attention in global health policy. The COVID-19 pandemic further exacerbated this challenge, as disruptions in routine care, reduced physical activity, and altered lifestyles likely increased population exposure to HFPG, aggravating the burden of related diseases. Disparities across socio-demographic (SDI) groups are also evident: low- and middle-income countries face steeper increases in HFPG burden due to weaker health systems and insufficient preventive measures, whereas some high-income countries have achieved partial control through screening and chronic disease management (10). Identifying high-risk populations and regions, along with evaluating the evolution of HFPG-attributable disease burden across different socioeconomic contexts, is essential for formulating effective and targeted global prevention strategies.

While prior studies have documented the global or regional burden of HFPG using earlier GBD data (2), significant gaps remain. First, none have integrated the latest GBD 2021 estimates with long-term projections to 2051 is lacking. Second, prior analyses often focus on a single dimension (e.g., global totals or specific diseases); a simultaneous examination of geographic, socio-demographic, age, and gender disparities, and their interplay, within a unified framework is needed to identify precise intervention targets. Third, the evolving gender disparity in burden distribution over three decades and its implications are underexplored. Therefore, this study aims not only to update the estimates but to provide a holistic, forward-looking analysis that quantifies inequalities and projects future trends, thereby offering actionable insights for stratified public health planning.

## Research design and methods

### Data sources

Data on the disease burden attributable to HFPG were sourced from the GBD 2021 study, published by the Institute for Health Metrics and Evaluation (IHME) (available at: https://ghdx.healthdata.org/gbd-2021) (11). This open-access dataset covers 204 countries and territories from 1990 to 2021 and provides epidemiological estimates for 371 diseases and 88 risk factors. Using the GBD Results Tool (https://vizhub.healthdata.org/gbd-results/), we extracted deaths, DALYs, mortality rates, and age-standardized DALY rates (ASDR) attributable to HFPG between 1990 and 2021.

### Definition of HFPG

For clinical context, HFPG is defined as a fasting plasma glucose level ≥5.6 mmol/L, which encompasses the range of impaired fasting glucose (prediabetes) and diabetes. In contrast, the TMREL, as defined by the GBD 2021 study, represents the level of fasting plasma glucose associated with the lowest risk of adverse health outcomes, set at 4.9–5.3 mmol/L. The primary analysis estimates the disease burden attributable to fasting plasma glucose levels exceeding this TMREL range. This estimates the preventable share linked to elevated glucose (including sub-diagnostic levels) and does not represent a distinct “HFPG-caused diabetes” subtype, but rather quantifies the etiological contribution of hyperglycemia to overall diabetes burden, consistent with established GBD methodology (1).

The disease burden attributable to HFPG encompasses 14 Level 3 causes for DALYs, including DM (T1DM and T2DM), IHD, stroke, CKD, Alzheimer's disease, and several cancers. Mortality estimates covered 13 causes, excluding blindness and vision loss (Supplementary Figure S1) (12).

### Data collection

The GBD modeling system employed DisMod-MR 2.1, a Bayesian meta-regression framework, to simulate health outcomes and integrate mortality data, generating initial estimates of HFPG-attributable mortality (13). DALYs, a composite measure of disease burden, combine years of life lost due to premature mortality and years lived with disability (14).

The SDI, a composite indicator of regional socioeconomic development—based on education, income, and fertility—was categorized into five quintiles (low, low-middle, middle, high-middle, high) across the 204 countries and territories included in GBD 2021 (15). Ranging from 0 to 1, the SDI enabled analysis of the association between HFPG-attributable disease burden—including age-standardized mortality rates (ASMR) and ASDR—and levels of socioeconomic development, thereby highlighting disparities in health impacts across different contexts.

### Statistical analysis

In this study, the disease burden attributable to HFPG was quantified using counts of deaths and DALYs, along with ASMR and ASDR. These indicators were further analyzed by age, sex, year, and geographic region. Age-standardized rates (ASR) were calculated based on the GBD 2021 global population age structure. To evaluate trends in ASMR and ASDR due to HFPG, we computed ASR per 100,000 population using the following formula (15):

$$ASR=\frac{\sum_{i=1}^{A}a_{i}w_{i}}{\sum_{i=1}^{A}w_{i}}\times 100,000$$

*a_i_* represents the age—specific rate for the *i-th* age group, *W* is the number of individuals in the corresponding age group of the standard population, and *A* is the number of age groups.

The estimated annual percentage change (EAPC) derived from a regression model characterizing the pattern of ASR over a specific period, with the equation Y = *α* + *β*X + e, where Y is the natural logarithm of ASR, X represents the years, α is the intercept, β is the slope or trend, and e is the error term (16). The EAPC is calculated as 100 × [exp(β) − 1], representing the annual percentage change. Trends were classified as increasing [EAPC and 95% uncertainty intervals (UI) > 0], decreasing (EAPC and 95% UI < 0), or stable.

A BAPC model with integrated nested Laplace Approximation (INLA), was used to forecast future trends in the global burden of disease attributable to HFPG up to 2051, with a focus on gender differences (16).

All statistical analyses were conducted using R version 4.4.1, with a *p*-value of less than 0.05 considered statistically significant.

## Results

### Global burden of disease attributable to HFPG

In absolute terms, the global number of deaths and DALYs attributable to HFPG increased substantially from 1990 to 2021. The detailed breakdown by gender is presented later. After age standardization, however, the rates remained relatively stable. In 2021, the global ASMR and ASDR attributable to HFPG were 63.73 (95% UI: 54.02–73.8) and 1,818.65 (95% UI: 1,540.62–2,098.68) per 100,000 population, respectively. From 1990 to 2021, the percentage changes were 0.02% (95% UI: −0.04 −0.07) for ASMR and 0.18% (95% UI: 0.12–0.24) for ASDR. While statistically stable, these minimal changes suggest that at the population level, the age-standardized risk of death and disability from HFPG has remained largely unchanged over the past three decades. The disease burden was consistently higher among males than females. DM was the leading Level 3 cause of HFPG-attributable burden globally, with an ASDR of 916.14 (95% UI: 776.14–1,096.55) and an ASMR of 19.61 (95% UI: 18.08–20.82) per 100,000. IHD and stroke followed, with ASDR/ASMR values of 303.49 (95% UI: 260.92–349.15)/16.27 (95% UI: 14.00–18.76) and 194.35 (95% UI: 150.47–239.28)/10.26 (95% UI: 8.03–12.56), respectively. In contrast, liver cancer showed the lowest burden, with an ASDR of 3.67 (95% UI: 0.41–7.36) and an ASMR of 0.17 (95% UI: 0.02–0.33). Notably, liver cancer exhibited the largest percentage increases in ASDR and ASMR from 1990 to 2021, rising by 0.61% (95% UI: 0.41–0.84) and 0.73% (95% UI: 0.51–0.96), respectively. Conversely, tuberculosis showed the most substantial decline, with ASDR and ASMR decreasing by −0.43% (95% UI: −0.51 to −0.26) and −0.45% (95% UI: −0.53 to −0.26), respectively (Table 1).

**Table 1:** Global burden of disease due to HFPG in 1990 and 2021. HFPG: high fasting plasma glucose.

Cause of death or DALYs	Mortality			DALYs		
	Age—standardized rate per 100,000 people (95%UI), in 1990	Age—standardized rate per 100,000 people (95%UI), in 2021	Percentage change in age—standardized rate from 1990 to 2021 (95%UI)	Age—standardized rate per 100,000 people (95%UI), in 1990	Age—standardized rate per 100,000 people (95%UI), in 2021	Percentage change in age—standardized rate from 1990 to 2021 (95%UI)
Global	62.71 (95%UI: 54.44, 71.24)	63.73 (95%UI: 54.02, 73.8)	0.02 (95%UI: −0.04, 0.07)	1,546.71 (95%UI: 1,330.59, 1,765.52)	1,818.65 (95%UI: 1,540.62, 2,098.68)	0.18 (95%UI: 0.12, 0.24)
Males	71.98 (95%UI: 63.00, 81.67)	73.86 (95%UI: 63.32, 85.23)	0.03 (95%UI: −0.04, 0.10)	1,755.41 (95%UI: 1,525.38, 1,998.89)	2,059.98 (95%UI: 1,757.45, 2,382.07)	0.17 (95%UI: 0.10, 0.25)
Females	55.80 (95%UI: 48.44, 63.76)	55.63 (95%UI: 46.48, 64.83)	0.00 (95%UI: −0.08, 0.07)	1,373.73 (95%UI: 1,179.76, 1,579.74)	1,606.12 (95%UI: 1,350.71, 1,864.70)	0.17 (95%UI: 0.10, 0.24)
Alzheimer's disease and other dementias	2.64 (95%UI: 0.11, 8.38)	3.73 (95%UI: 0.15, 11.84)	0.41 (95%UI: 0.33, 0.51)	47.07 (95%UI: 2.72, 126.46)	66.42 (95%UI: 3.83, 178.85)	0.41 (95%UI: 0.34, 0.48)
Bladder cancer	0.18 (95%UI: −0.02, 0.4)	0.21 (95%UI: −0.03, 0.48)	0.21 (95%UI: 0.11, 0.35)	3.29 (95%UI: −0.44, 7.44)	3.81 (95%UI: −0.49, 8.64)	0.16 (95%UI: 0.07, 0.31)
Blindness and vision loss	/	/	/	16.95 (95%UI: −2.22, 40.83)	19.72 (95%UI: −2.51, 46.03)	0.16 (95%UI: 0.08, 0.27)
Breast cancer	0.29 (95%UI: −0.08, 0.68)	0.36 (95%UI: −0.1, 0.85)	0.23 (95%UI: 0.15, 0.32)	7.33 (95%UI: −2.13, 17.25)	9.43 (95%UI: −2.77, 22.26)	0.29 (95%UI: 0.2, 0.39)
Chronic kidney disease	4.99 (95%UI: 3.15, 7.03)	7.95 (95%UI: 5.17, 10.65)	0.59 (95%UI: 0.39, 0.78)	127.88 (95%UI: 78.92, 182.88)	187.46 (95%UI: 123.18, 257.57)	0.47 (95%UI: 0.31, 0.63)
Colon and rectum cancer	0.89 (95%UI: 0.45, 1.34)	0.98 (95%UI: 0.51, 1.49)	0.1 (95%UI: 0.02, 0.1)	18.46 (95%UI: 9.27, 28.03)	20.31 (95%UI: 10.46, 30.81)	0.1 (95%UI: 0.01, 0.2)
Diabetes mellitus	18.17 (95%UI: 17.02, 19.06)	19.61 (95%UI: 18.08, 20.82)	0.08 (95%UI: 0.01, 0.15)	662.99 (95%UI: 586.94, 761.59)	916.14 (95%UI: 776.14, 1096.55)	0.38 (95%UI: 0.3, 0.47)
Ischemic heart disease	17.08 (95%UI: 14.59, 19.76)	16.27 (95%UI: 14, 18.76)	−0.05 (95%UI: −0.1, 0)	304.98 (95%UI: 259.97, 351.32)	303.49 (95%UI: 260.92, 349.15)	0 (95%UI: −0.06, 0.05)
Liver cancer	0.1 (95%UI: 0.01, 0.2)	0.17 (95%UI: 0.02, 0.33)	0.73 (95%UI: 0.51, 0.96)	2.28 (95%UI: 0.24, 4.65)	3.67 (95%UI: 0.41, 7.36)	0.61 (95%UI: 0.41, 0.84)
Lower extremity peripheral arterial disease	0.28 (95%UI: 0.21, 0.35)	0.32 (95%UI: 0.25, 0.39)	0.15 (95%UI: 0.07, 0.25)	5.61 (95%UI: 4.12, 7.71)	6.73 (95%UI: 5.06, 9.23)	0.2 (95%UI: 0.12, 0.29)
Pancreatic cancer	1.08 (95%UI: 0.12, 2.11)	1.57 (95%UI: 0.18, 2.98)	0.45 (95%UI: 0.34, 0.57)	22.64 (95%UI: 2.58, 44.6)	31.7 (95%UI: 3.63, 59.9)	0.4 (95%UI: 0.28, 0.54)
Stroke	13.41 (95%UI: 10.51, 16.55)	10.26 (95%UI: 8.03, 12.56)	−0.24 (95%UI: −0.29, −0.17)	240.18 (95%UI: 186.21, 299.18)	194.35 (95%UI: 150.47, 239.28)	−0.19 (95%UI: −0.25, −0.12)
Tracheal, bronchus, and lung cancer	0.49 (95%UI: −0.1, 1.1)	0.59 (95%UI: −0.12, 1.35)	0.19 (95%UI: 0.06, 0.32)	11.33 (95%UI: −2.34, 25.05)	12.43 (95%UI: −2.48, 28.54)	0.1 (95%UI: −0.03, 0.22)
Tuberculosis	3.1 (95%UI: 2.22, 4.1)	1.72 (95%UI: 1.25, 2.29)	−0.45 (95%UI: −0.53, −0.26)	75.73 (95%UI: 54.56, 100.06)	42.99 (95%UI: 31.17, 57.18)	−0.43 (95%UI: −0.51, −0.26)

Diseases are listed in alphabetical order. The three leading causes are discussed in the main text.

### Region burden of disease attributable to HFPG

In terms of absolute burden, the most populous regions carried the largest number of cases. However, when examining ASR, North Africa and the Middle East, along with Oceania, exhibited the highest disease burden attributable to HFPG in both 1990 and 2021, particularly for colorectal cancer and DM. In 2021, the North Africa and Middle East region recorded the highest regional ASMR for IHD at 38.42 (95% UI: 32.30–45.39) and the highest ASDR for DM at 1,344.82 (95% UI: 1,115.75–1,672.17) per 100,000. In the same year, Oceania reported an ASMR and ASDR for DM of 109.64 (95% UI: 93.65–130.35) and 3,585.13 (95% UI: 3,067.16–4,154.64) per 100,000, respectively (Supplementary Figure S2).

Marked gender and age disparities were observed in the HFPG-attributable burden of IHD and stroke. In high-income North America, the highest ASDR for IHD occurred among males (21.00%), while among females, it peaked in the North Africa and Middle East (19.00%) (Supplementary Figure S3A). For stroke, the highest ASDR for both sexes was observed in North Africa and the Middle East, with rates of 17.00% in males and 16.00% in females (Supplementary Figure S3B). Age-specific analysis revealed a progressive increase in the stroke burden, peaking in the 95+ age group—14.70% for males and 13.99% for females (Supplementary Figure S3D). In contrast, the IHD burden peaked in the 75–79 age group, reaching 17.32% in males and 16.52% in females (Supplementary Figure S3C).

### Global burden of disease attributable to HFPG across 204 countries and territories

**Figure 1:**
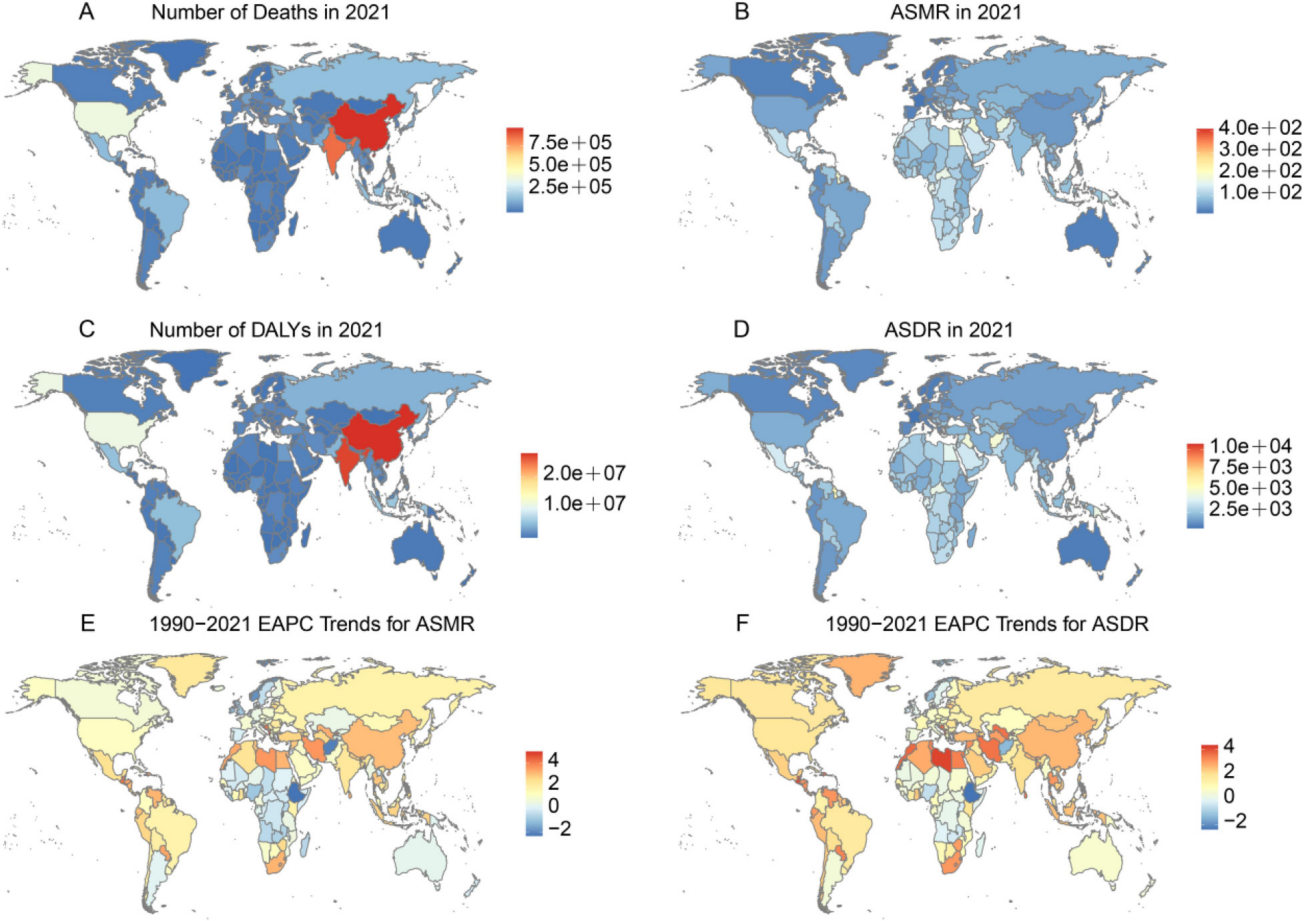
The burden of disease attributable to HFPG for deaths, DALYs cases, mortality, DALYs rates in 2021, and EAPC for ASMR and ASDR from 1990 to 2021, across 204 countries and territories. **(A)** Number of deaths in 2021. **(B)** ASMR in 2021. **(C)** Number of DALYs in 2021. **(D)** ASDR in 2021. **(E)** EAPC for ASMR from 1990 to 2021. **(F)** EAPC for ASDR from 1990 to 2021. EAPC: estimated annual percentage change. Maps generated using the R package https://cran.r-project.org/web/packages/maps/index.html by Richard A. Becker, Allan R. Wilks, Ray Brownrigg, Thomas P. Minka and Alex Deckmyn, licensed under GNU General Public License, version 2.

Regarding absolute counts, China reported the highest numbers of HFPG-attributable deaths and DALYs in both 1990 and 2021 (Figure 1, Supplementary Figure S4). In 2021, China recorded 956,264.44 deaths (95% UI: 759, 956.10–1,188,899.20) and 27,655,530.55 DALYs (95% UI: 22,285, 715.95–33,178, 876.80). Tokelau reported the lowest numbers, with 3.14 deaths (95% UI: 2.55–3.86) and 89.76 DALYs (95% UI: 74.79–107.87). In contrast, based on ASR, The Marshall Islands had the highest ASMR and ASDR, at 10,691.10 (95% UI: 8,482.36–13,433.88) and 7,927.59 (95% UI: 6,801.33–9,043.47) per 100,000, respectively. From 1990 to 2021, Albania experienced the largest estimated annual percentage change (EAPC) in ASMR and ASDR, at 5.04 (95% UI: 4.80–5.28) and 4.37 (95% UI: 4.22–4.52), respectively. Conversely, Ethiopia showed the lowest EAPC, with −2.31 (95% UI: −2.61 to −2.00) for ASMR and −2.38 (95% UI: −2.68 to −2.08) for ASDR (Figure 1).

### The burden of disease attributable to HFPG by SDI

**Figure 2:**
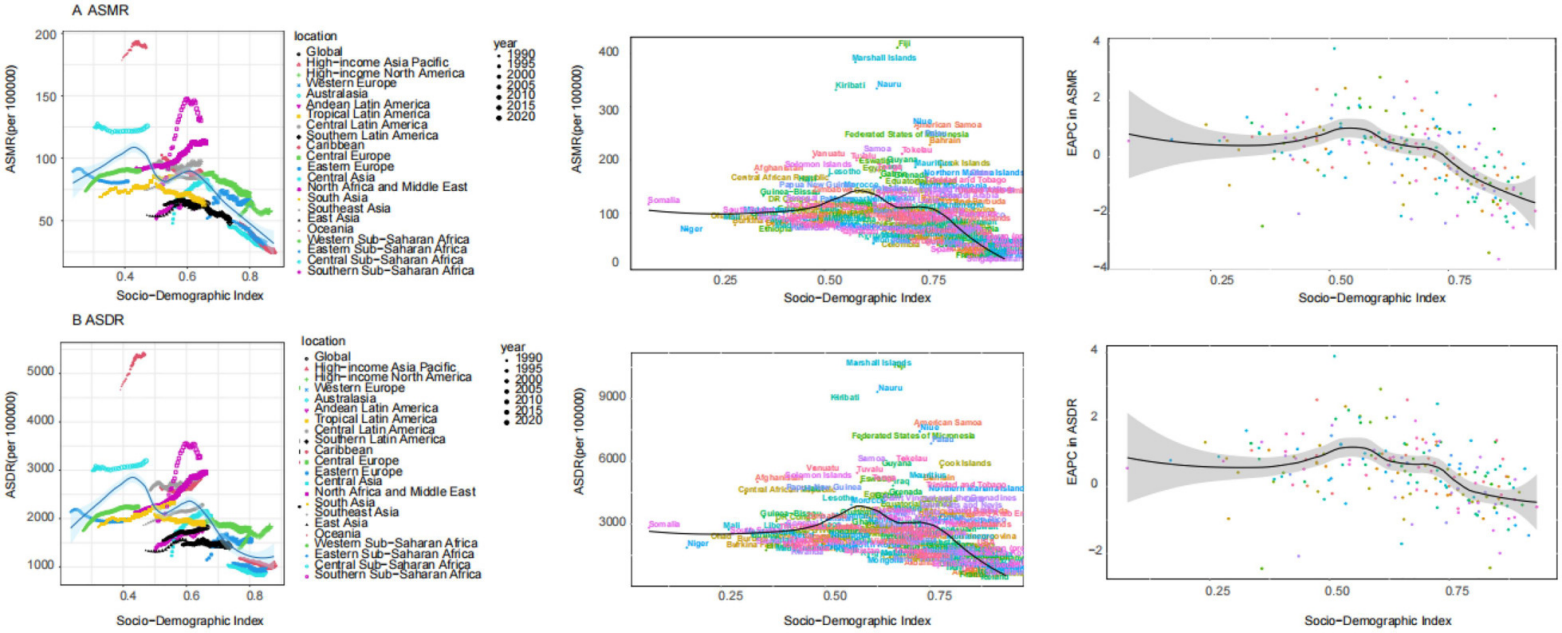
The burden of disease attributable to HFPG across 21 GBD regions, across 204 countries and territories, and EAPC by SDI. **(A)** ASMR. **(B)** ASDR.

The relationship between HFPG-attributable disease burden and the SDI showed a non-linear trend. Both ASMR and ASDR peaked at moderate SDI levels (approximately 0.56–0.60), then declined at higher SDI levels, suggesting that higher socioeconomic development is generally associated with a lower disease burden. Notably, Oceania and sub-Saharan Africa exhibited higher ASMR and ASDR than other regions at similar SDI levels. Across the 204 countries and territories in 2021, ASMR and ASDR initially increased with SDI, reaching a peak around SDI 0.60 before declining. Countries such as Fiji, the Marshall Islands, Nauru, and Kiribati reflected this trend, showing relatively high disease burdens at moderate SDI levels. In contrast, in countries with SDI greater than 0.75, the EAPC of ASMR and ASDR declined, indicating a downward trend in HFPG-attributable burden as SDI increased (Figure 2).

### Gender- and age-specific global burden of disease attributable to HFPG

**Figure 3:**
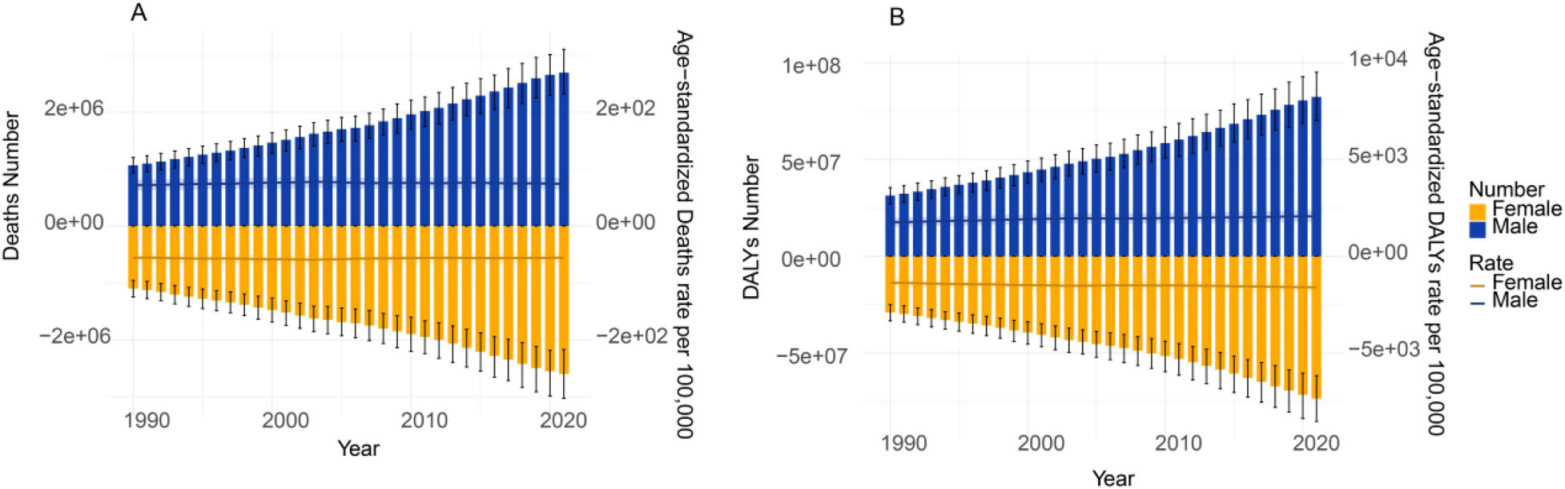
Deaths, number of DALYs, ASMR, and ASDR for the burden of disease due to HFPG by sex, 1990–2021. **(A)** Deaths and ASMR. **(B)** number of DALYs and ASDR.

In absolute terms, the global number of deaths and DALYs attributable to HFPG showed a consistent upward trend from 1990 to 2021, with males consistently experiencing a higher burden than females (Figure 3). Specifically, the number of deaths among males increased from 1,065,990.24 (95% UI: 934,322.31–1,204,677.24) to 2,694,214.60 (95% UI: 2,318,880.83–3,103,819.32), representing a 2.53-fold increase, while DALYs rose from 31,318,723.08 (95% UI: 27,085,191.87–35,463,134.88) to 82,265,037.85 (95% UI: 70,175,025.59–94,993,881.94), a 2.63-fold increase. Among females, deaths increased from 1,097,073.23 (95% UI: 957,810.06–1,248,702.32) to 2,598,612.31 (95% UI: 2,170,140.15–3,030,576.07), a 2.37-fold rise, and DALYs climbed from 29,028,417.27 (95% UI: 24,997,634.77–33,340,633.39) to 73,417,208.56 (95% UI: 61,686,107.51–85,262,053.28), also a 2.53-fold increase. In contrast, ASR remained relatively stable over the same period. For instance, the global ASDR increased only marginally, from 1,765.52 to 2,098.68 per 100,000 population. Notably, the gender distribution of the disease burden shifted markedly over the 31-year period. In terms of mortality share, although females accounted for a slightly higher proportion in 1990 (50.72% vs. 49.28% for males), this pattern reversed over time. By 2021, the male share of deaths had risen to 50.90%, while the female share decreased to 49.10%. Male predominance was even more pronounced in the distribution of DALYs. In 1990, males already accounted for 51.90% of global HFPG-attributable DALYs, compared to 48.10% for females. This male predominance further intensified, with the male share of DALYs peaking at 53.18% in 2011 and remaining high at 52.84% by 2021.

**Figure 4:**
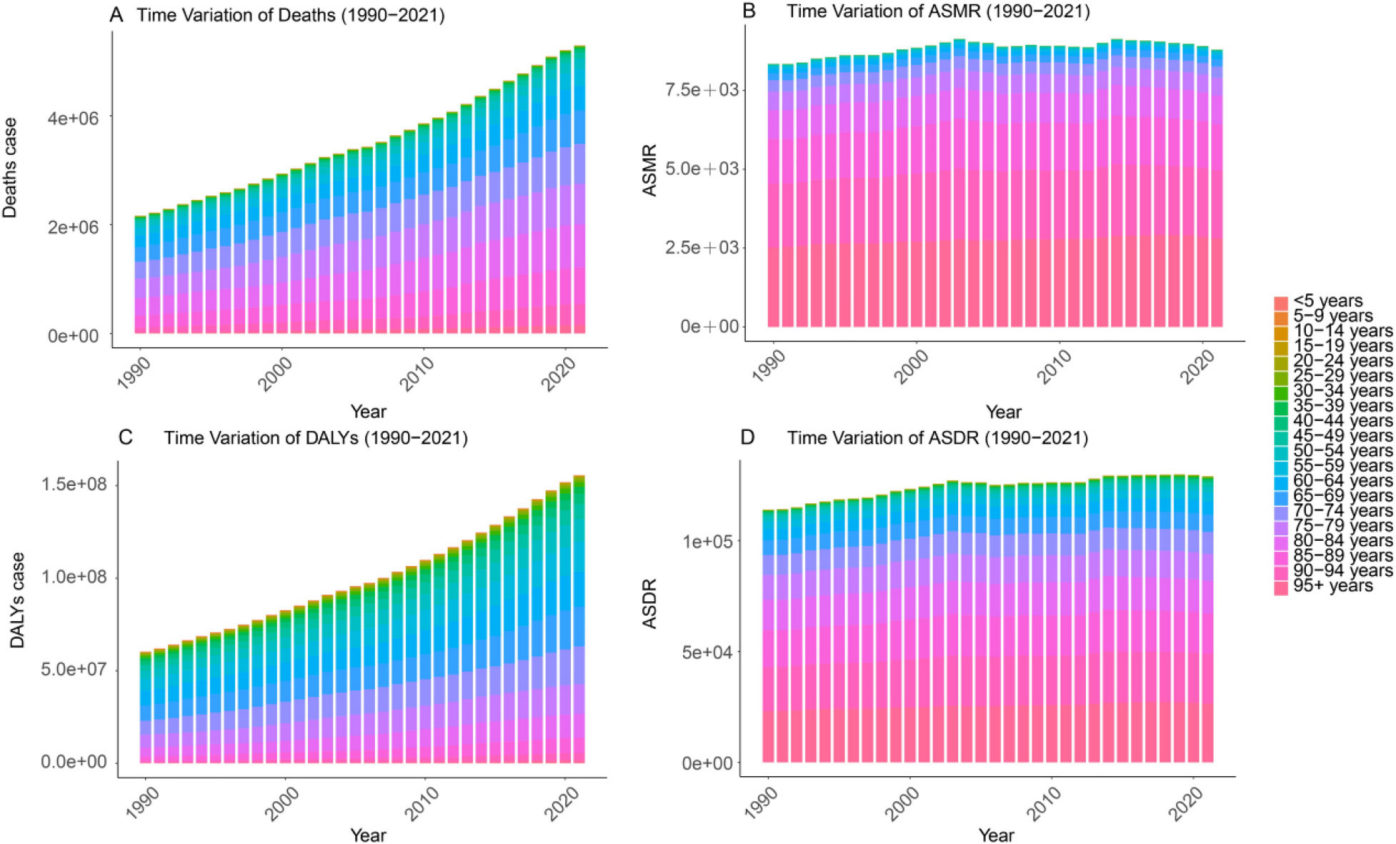
Deaths **(A)**, number of DALYs **(C)**, ASMR **(B)**, and ASDR **(D)** for the burden of disease due to HFPG by age from 1990 to 2021. **(A)** Deaths. **(B)** ASMR. **(C)** DALYs. **(D)** ASDR.

The disease burden attributable to HFPG increased across most age groups, with the most pronounced rise observed among middle-aged and older adults (Figure 4).

In 2021, the highest numbers of deaths occurred in the 80–84 and 75–79 age groups, with 799,870.96 (95% UI: 677,939.24–942,428.76) and 744,283.07 (95% UI: 638,002.46–857,855.15) cases, accounting for 15.11% and 14.06% of total deaths, respectively. In contrast, the 5–9 year age group had the lowest number of deaths, at 893.21 (95% UI: 616.33–1,091.74), representing only 0.01% of the total. In terms of disability-adjusted life years (DALYs), the 65–69 year age group carried the highest burden, with 21,446,301.19 (95% UI: 18,212,921.51–24,676,934.97) DALYs, comprising 13.77% of the total. The lowest DALY burden was observed in the 5–9 year age group, at 100,368.60 (95% UI: 73,740.83–123,592.87), which accounted for 0.06% of total DALYs.

### Projections for the global burden of disease attributable to HFPG

**Figure 5:**
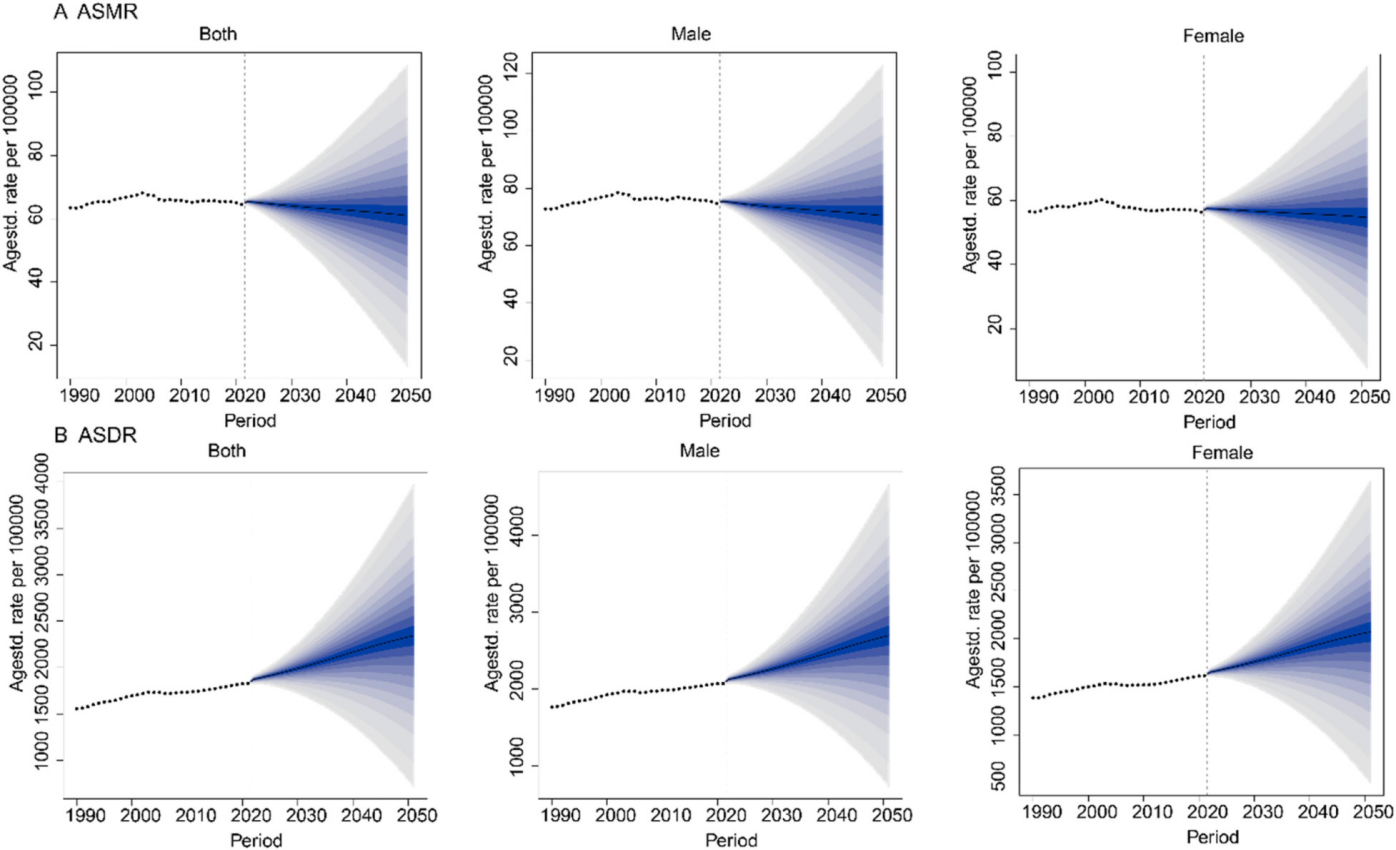
The ASMR and ASDR for diseases due to HFPG projected to 2051. **(A)** ASMR. **(B)** ASDR.

Using the BAPC model, we projected the global disease burden attributable to HFPG from 2022 to 2051 (Figure 5). The results indicate a decline in ASMR over this period. By 2051, the global ASMR is projected to reach 60.97 per 100,000 population (95% UI: 36.61–85.33). The ASMR for males is projected to be 70.46 (95% UI: 43.58–97.35), compared to 54.71 (95% UI: 30.66–78.76) for females. In contrast, the global ASDR is projected to increase, reaching 2,343.46 per 100,000 population (95% UI: 1,511.86–3,175.07) by 2051. The ASDR for males is projected to be 2,696.87 (95% UI: 1,694.48–3,699.26), while for females, it is expected to be 2,070.81 (95% UI: 1,267.36–2,874.27). The wide uncertainty intervals reflect the inherent challenge of long-term forecasting; however, the central trend of declining ASMR and rising ASDR provides a robust strategic warning for health system planning.

## Discussion

In 2021, DM remained the primary contributor to the global disease burden attributable to HFPG, with an ASDR of 916.14 per 100,000 population (95% UI: 776.14–1096.55) and an ASMR of 19.61 per 100,000 population (95% UI: 18.08–20.82). IHD and stroke were the next major contributors. These findings are consistent with growing evidence on the impact of metabolic disorders on global health (17). Although ASMR has shown a decline, ASDR continues to rise, indicating that while premature mortality is being reduced, the burden of chronic HFPG-related conditions such as diabetes and cardiovascular diseases is increasing. This trend may reflect improvements in clinical management and early detection, which have prolonged life expectancy but also resulted in more individuals living with chronic diseases. This pattern aligns with the global shift toward chronic non-communicable diseases, particularly type 2 diabetes (13, 18, 19), and supports the theory of epidemiological transition.

Significant geographic disparities were observed in the HFPG-attributable burden, with the highest ASMR and ASDR seen in North Africa and the Middle East, as well as Oceania. These regions are experiencing rapid lifestyle transitions characterized by accelerated urbanization, increased consumption of high-calorie diets, and more sedentary behaviors. At the same time, many of these areas lack strong primary prevention systems and public health infrastructure, further exacerbating the HFPG-related disease burden (20). Conversely, the relatively lower ASMR and ASDR in high-income regions such as North America and Australia may reflect more advanced healthcare systems, earlier diagnosis, and better management of diabetes and its cardiovascular complications, underscoring the potential for health system improvements to mitigate burden. These findings reinforce earlier evidence that economic development without concurrent strengthening of health systems can worsen the burden of non-communicable diseases.

At the national level, China accounted for the largest number of HFPG-related deaths cases (956,264; 95% UI: 759,956–1,188,899) and DALYs cases (27,655,531; 95% UI: 22,285,716–33,178,877), which can be attributed to its large population, aging demographic structure, and rising prevalence of diabetes (21–23). In contrast, the Marshall Islands reported the highest ASMR (10,691.1 per 100,000; 95% UI: 8,482.4–13,433.9) and ASDR (7,927.6 per 100,000; 95% UI: 6,801.3–9,043.5), reflecting the disproportionate burden in small island nations with limited healthcare capacity and chronic disease management. This illustrates the dual challenge in many low- and middle-income countries, where the increasing prevalence of metabolic conditions is met with insufficient healthcare resources (2, 24).

The HFPG-attributable burden was consistently higher among males, who exhibited significantly elevated ASMR and ASDR compared to females. The observed population-level disparity suggests the potential influence of factors such as biological susceptibility, differential exposure to behavioral risk factors (e.g., smoking, alcohol consumption), or healthcare utilization patterns, which require individual-level studies to confirm (2, 24). Furthermore, the burden demonstrated a steep age gradient, with IHD peaking in the 75–79 age group and stroke burden highest among those aged 95 and above. These patterns underscore the cumulative effects of metabolic dysregulation over the life course (11, 25) and are consistent with emerging evidence on “metabolic memory” and the long-term complications of hyperglycemia (11, 26, 27).

The relationship between HFPG burden and the SDI was nonlinear. The highest burden occurred in countries with moderate SDI levels (0.56–0.6), whereas high-SDI countries showed declining trends. This pattern reflects the “risk transition” theory, whereby economic development precedes the maturation of healthcare systems. Without parallel investments in public health infrastructure and health education, the burden of metabolic diseases tends to increase during this transitional period. This trend is especially pronounced in countries experiencing rapid Westernization of lifestyles, increased sedentary behavior, and unhealthy dietary patterns (28, 29). Notably, small island nations such as the Marshall Islands were identified as extreme outliers, consistent with studies highlighting the combined effects of nutrition transition and limited health services (28, 29).

Projections based on the BAPC model suggest that while global ASMR will continue to decline in the coming decades, ASDR will keep rising. This indicates that although acute mortality may be reduced through clinical management, the total burden is likely to grow due to global population aging and expansion. These findings emphasize the need for public health systems to shift focus from merely reducing mortality to minimizing disability and enhancing quality of life. Middle SDI countries in particular should prioritize early screening, lifestyle interventions, and long-term chronic disease management to mitigate the long-term impact of HFPG. Evidence-based strategies such as community—based diabetes prevention programs (30) and sugar-sweetened beverage taxes (30) have shown success and could be adapted for implementation in high-burden regions. The growing disability burden also highlights the urgent need to expand rehabilitation services, consistent with the WHO's Rehabilitation 2030 initiative (2).

This study extends prior research by employing the most recent GBD 2021 data and projecting trends to 2051, providing an updated, forward-looking perspective that highlights the impending growth in ASDR alongside stable mortality rates. Our integration of SDI, age, and gender analyses reveals that the highest burden clusters in middle-SDI countries and identifies males and older adults as persistently high-risk groups, including a documented reversal in gender mortality share over 31 years. These findings, which pinpoint specific high-burden geographic and demographic hotspots, translate into clear, actionable priorities: targeted screening and early management of elevated fasting glucose (especially in the prediabetes range) in high-risk populations and regions; strengthening integrated chronic care and medication access to address the growing disability burden; and focused policy and investment in health systems of middle-SDI countries and small island nations where systemic gaps and demographic trends amplify the burden. While this comprehensive, population-level assessment prioritizes identifying broad inequalities and future trends over deep-dive mechanistic exploration, it establishes a crucial evidence base for prioritizing targeted, in-depth studies and resource allocation. However, several limitations must be noted. First, Our finding that diabetes mellitus is the leading contributor to the HFPG-attributable disease burden requires careful interpretation to prevent misunderstanding. As outlined in the Methods, this result should be viewed through the lens of population-attributable risk. The large burden estimate underscores that a substantial portion of the global diabetes epidemic is etiologically linked to elevated fasting plasma glucose, including levels in the pre-diabetes and diabetes ranges. For clinicians and policymakers, this emphasizes that interventions aimed at lowering population-wide glucose levels (e.g., through dietary, physical activity, and weight management programs) could potentially prevent a significant share of future diabetes cases, in line with the TMREL concept. It does not imply the existence of a separate “HFPG-diabetes” category in clinical practice, but rather highlights the dominant role of hyperglycemia in the diabetes disease pathway at a population level. Second, FPG data were lacking for several countries or territories, potentially affecting estimate precision. Third, Our projections of future HFPG-attributable burden using the BAPC model are based on the continuation of past epidemiological and demographic trends. While this offers a valuable baseline scenario, it inherently does not account for potential future changes in clinical practice, pharmacotherapy, intensified screening programs, or major policy interventions aimed at diabetes prevention and control. Consequently, the projected divergence between declining ASMR and rising ASDR should be interpreted with caution. This pattern likely reflects the dominant influence of global population aging—leading to more people living longer with chronic, disabling conditions—rather than a necessary worsening of metabolic risk at the individual level. In reality, advances in treatment and prevention could moderate the rise in ASDR, while improvements in acute care may further reduce ASMR. Thus, our projections illustrate a demography-driven trajectory under current trends, and they highlight the urgent need for innovative clinical and public health strategies to alter this course. Furthermore, our analysis is based on national-level ecological data. While it reveals important population-level associations and trends, it cannot establish causality or directly infer individual-level risk mechanisms. Explanations for observed disparities (e.g., by sex or region) should therefore be interpreted as plausible hypotheses generated from the aggregate data and the broader literature, rather than conclusions drawn from the present study design. Finally, this analysis used ecological data at the national level, which may mask subnational disparities and introduce misclassification or ecological bias.

## Conclusions

DM, IHD, and stroke remain the primary contributors to the global disease burden attributable to HFPG. Significant geographic disparities persist, with North Africa and the Middle East, along with Oceania, experiencing disproportionately high impacts. Notable demographic inequalities are also evident, as males and older adults continue to bear a substantially greater burden.

Projections to 2051 indicate a paradoxical trend: while the ASMR is expected to decline, the ASDR is projected to rise. This pattern reflects an expanding absolute burden, driven largely by population aging and growth worldwide.

Addressing this challenge requires urgent and targeted interventions, particularly in regions with middle SDI levels and among high-risk demographic groups. Mitigating the long-term health and economic impacts of HFPG will depend on prioritizing integrated primary and secondary prevention strategies within broader chronic disease frameworks and strengthening health systems in the most vulnerable regions. These actions should form a cornerstone of the global public health agenda.

## Data Availability

Publicly available datasets were analyzed in this study. This data can be found here: Global Burden of Disease (GBD) https://ghdx.healthdata.org/gbd-2021.

## References

[1] GBD 2021 Risk Factors Collaborators. Global burden and strength of evidence for 88 risk factors in 204 countries and 811 subnational locations, 1990–2021: a systematic analysis for the global burden of disease study 2021. Lancet. (2024) 403(10440):2162–203. 10.1016/S0140-6736(24)00933-4 38762324 PMC11120204

[2] Liang R Feng X Shi D Yang M Yu L Liu W The global burden of disease attributable to high fasting plasma glucose in 204 countries and territories, 1990–2019: an updated analysis for the global burden of disease study 2019. Diabetes-Metab Res Rev. (2022) 38(8):e3572. 10.1002/dmrr.3572 36001650

[3] Chew NWS Ng CH Tan DJH Kong G Lin C Chin YH The global burden of metabolic disease: data from 2000 to 2019. Cell Metab. (2023) 35(3):414–28. 10.1016/j.cmet.2023.02.003 36889281

[4] Zheng Y Ley SH Hu FB. Global aetiology and epidemiology of type 2 diabetes mellitus and its complications. Nat Rev Endocrinol. (2018) 14(2):88–98. 10.1038/nrendo.2017.151 29219149

[5] Sun H Saeedi P Karuranga S Pinkepank M Ogurtsova K Duncan BB IDF Diabetes atlas: global, regional and country-level diabetes prevalence estimates for 2021 and projections for 2045. Diabetes Res Clin Pract. (2022) 183:109119. 10.1016/j.diabres.2021.109119 34879977 PMC11057359

[6] Russell NDF Cooper ME. 50 Years forward: mechanisms of hyperglycaemia-driven diabetic complications. Diabetologia. (2015) 58(8):1708–14. 10.1007/s00125-015-3600-1 25906755

[7] GBD 2019 Diabetes in the Americas Collaborators. Burden of diabetes and hyperglycaemia in adults in the americas, 1990–2019: a systematic analysis for the global burden of disease study 2019. Lancet Diabetes Endocrinol. (2022) 10(9):655–67. 10.1016/S2213-8587(22)00186-3 35850129 PMC9399220

[8] Cao X Tian Y Zhao Z Wang L Wang X Zheng C Disparities in high fasting plasma glucose-related cardiovascular disease burden in China. Nat Commun. (2024) 15(1):8817. 10.1038/s41467-024-53236-y 39394204 PMC11470015

[9] Zheng Z Xu S Zhu J Yang Q Ye H Li M Disease burden of cancers attributable to high fasting plasma glucose from 1990 to 2021 and projections until 2031 in China. Cancer Epidemiol. (2025) 94:102725. 10.1016/j.canep.2024.102725 39708577

[10] Zhang H Zhou X Shapiro MD Lip GYH Tilg H Valenti L Global burden of metabolic diseases, 1990–2021. Metab, Clin Exp. (2024) 160:155999. 10.1016/j.metabol.2024.155999 39151887

[11] Dong X Zhang X Wang B Hou F Jiao Y. The burden of cardiovascular disease attributable to high fasting plasma glucose: findings from the global burden of disease study 2019. Diabetes Metab Syndr Clin Res Rev. (2024) 18(5):103025. 10.1016/j.dsx.2024.103025 38851022

[12] GBD 2019 Diseases and Injuries Collaborators. Global burden of 369 diseases and injuries in 204 countries and territories, 1990–2019: a systematic analysis for the global burden of disease study 2019. Lancet. (2020) 396(10258):1204–22. 10.1016/S0140-6736(20)30925-9 33069326 PMC7567026

[13] Gebeyehu DT East L Wark S Islam MS. Disability-adjusted life years (DALYs) based COVID-19 health impact assessment: a systematic review. BMC Public Health. (2023) 23(1):334. 10.1186/s12889-023-15239-0 36793006 PMC9929217

[14] Sanjamala HSR Singhvi M Shah PA. Socio-demographic index and socioeconomic classes for understanding the divisible differences in receiving multimodal therapy in patients with pancreatic cancers. J Surg Oncol. (2023) 127(1):207–8. 10.1002/jso.27099 36330580

[15] Gandaglia G Leni R Bray F Fleshner N Freedland SJ Kibel A Epidemiology and prevention of prostate cancer. Eur Urol Oncol. (2021) 4(6):877–92. 10.1016/j.euo.2021.09.006 34716119

[16] Wang R Li Z Liu S Zhang D. Global, regional and national burden of inflammatory bowel disease in 204 countries and territories from 1990 to 2019: a systematic analysis based on the global burden of disease study 2019. BMJ Open. (2023) 13(3):e65186. 10.1136/bmjopen-2022-065186 PMC10069527 36977543

[17] GBD 2021 Diabetes Collaborators. Global, regional, and national burden of diabetes from 1990 to 2021, with projections of prevalence to 2050: a systematic analysis for the global burden of disease study 2021. Lancet. (2023) 402(10397):203–34. 10.1016/S0140-6736(23)01301-6 37356446 PMC10364581

[18] El-Kebbi IM Bidikian NH Hneiny L Nasrallah MP. Epidemiology of type 2 diabetes in the Middle East and north Africa: challenges and call for action. World J Diabetes. (2021) 12(9):1401–25. 10.4239/wjd.v12.i9.1401 34630897 PMC8472500

[19] Ma Y Huang S Dong Y Jin Q. Global burden of Alzheimer’s disease attributable to high fasting plasma glucose: epidemiological trends and machine learning insights. Risk Manag Healthc Policy. (2025) 18:1291–307. 10.2147/RMHP.S506581 40255880 PMC12007509

[20] Song L Chen Z Li Y Ran L Liao D Zhang Y Trend and forecast analysis of the changing disease burden of pancreatic cancer attributable to high fasting glucose in China, 1990–2021. Front Oncol. (2024) 14:1471699. 10.3389/fonc.2024.1471699 39493456 PMC11527594

[21] Deng W Zhao L Chen C Ren Z Jing Y Qiu J National burden and risk factors of diabetes mellitus in China from 1990 to 2021: results from the global burden of disease study 2021. J Diabetes. (2024) 16(10):e70012. 10.1111/1753-0407.70012 39373380 PMC11457207

[22] Safiri S Nejadghaderi SA Karamzad N Kaufman JS Carson-Chahhoud K Bragazzi NL Global, regional and national burden of cancers attributable to high fasting plasma glucose in 204 countries and territories, 1990–2019. Front Endocrinol (Lausanne). (2022) 13:879890. 10.3389/fendo.2022.879890 35966097 PMC9366927

[23] Feng G Targher G Byrne CD Yilmaz Y Wai-Sun Wong V Adithya Lesmana CR Global burden of metabolic dysfunction-associated steatotic liver disease, 2010 to 2021. JHEP Reports. (2025) 7(3):101271. 10.1016/j.jhepr.2024.101271 39980749 PMC11840544

[24] Yang G Au Yeung SL Schooling CM. Sex differences in the association of fasting glucose with HbA1c, and their consequences for mortality: a Mendelian randomization study. EBioMedicine. (2022) 84:104259. 10.1016/j.ebiom.2022.104259 36179552 PMC9520189

[25] Wang W Hu M Liu H Zhang X Li H Zhou F Global burden of disease study 2019 suggests that metabolic risk factors are the leading drivers of the burden of ischemic heart disease. Cell Metab. (2021) 33(10):1943–56. 10.1016/j.cmet.2021.08.005 34478633

[26] Zhang K Ma Y Luo Y Song Y Xiong G Ma Y Metabolic diseases and healthy aging: identifying environmental and behavioral risk factors and promoting public health. Front Public Health. (2023) 11:1253506. 10.3389/fpubh.2023.1253506 37900047 PMC10603303

[27] Richards SE Wijeweera C Wijeweera A. Lifestyle and socioeconomic determinants of diabetes: evidence from country-level data. PLoS One. (2022) 17(7):e270476. 10.1371/journal.pone.0270476 PMC9333224 35901054

[28] Forray AI Borzan CM. Implementation of national nutrition policies and strategies to reduce unhealthy diets: an ecological analysis of 194 countries from 2017 to 2021. Nutrients. (2024) 16(6):911. 10.3390/nu16060911 38542822 PMC10975650

[29] Teng AM Jones AC Mizdrak A Signal L Genc M Wilson N. Impact of sugar-sweetened beverage taxes on purchases and dietary intake: systematic review and meta-analysis. Obes Rev. (2019) 20(9):1187–204. 10.1111/obr.12868 31218808 PMC9285619

[30] Cieza A Causey K Kamenov K Hanson SW Chatterji S Vos T. Global estimates of the need for rehabilitation based on the global burden of disease study 2019: a systematic analysis for the global burden of disease study 2019. Lancet. (2021) 396(10267):2006–17. 10.1016/S0140-6736(20)32340-0 33275908 PMC7811204

