# Supplementary material for: Global burden of disease attributable to high fasting plasma glucose from 1990 to 2021: a spatiotemporal analysis of global burden of disease 2021
Source: Front Cardiovasc Med. 2026 Feb 17;13:1679255. doi: 10.3389/fcvm.2026.1679255 (PMC12953428; doi:10.3389/fcvm.2026.1679255)
Supplement: Supplementary file 1 [file Table1.docx]

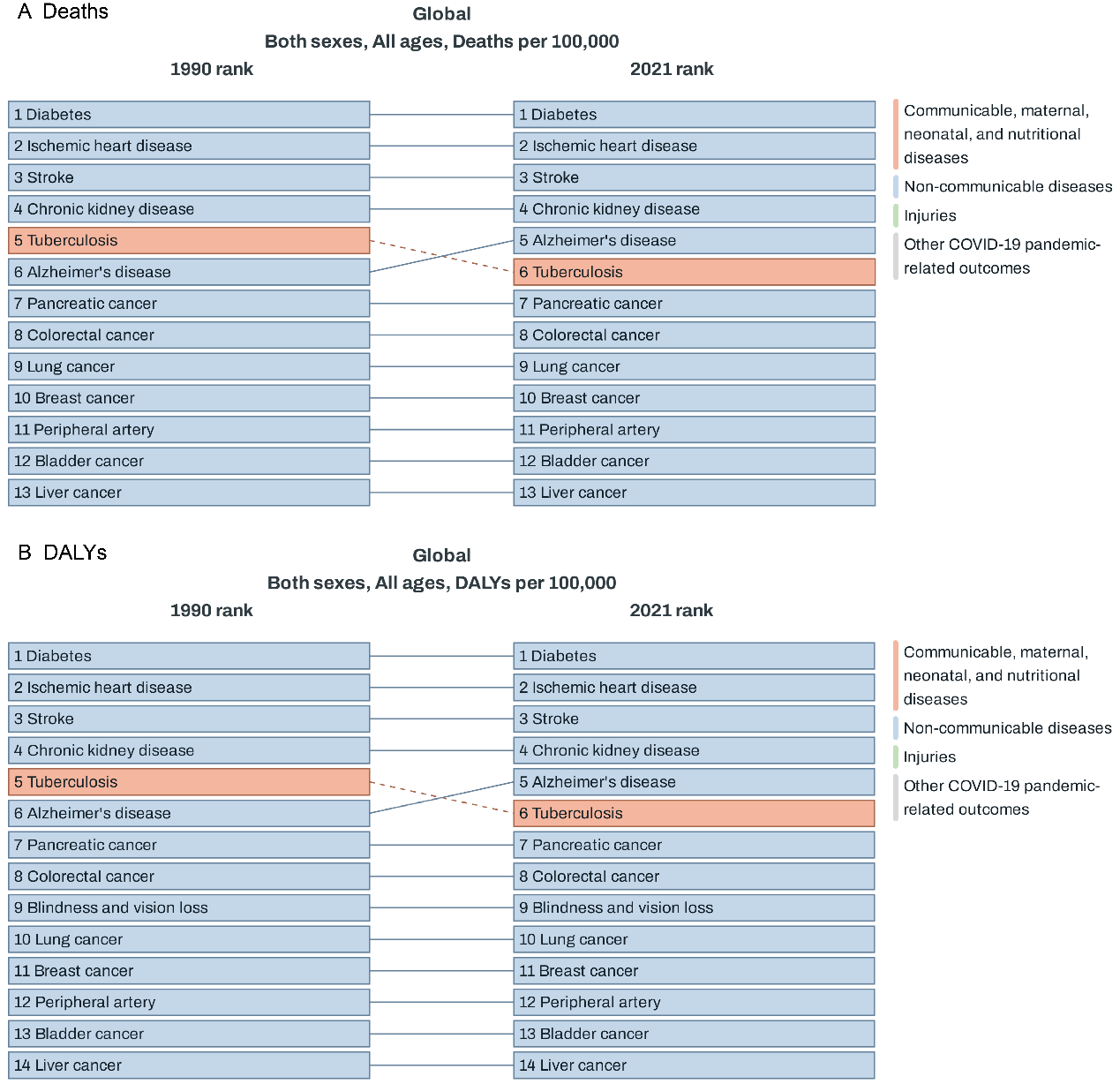


Figure S1 The GBD 2021 analysis of diseases attributable to high fasting plasma glucose. (A) Deaths are associated with 13 types of diseases at the 3 levels. (B) DALYs are associated with 14 types of diseases at the 3 levels.


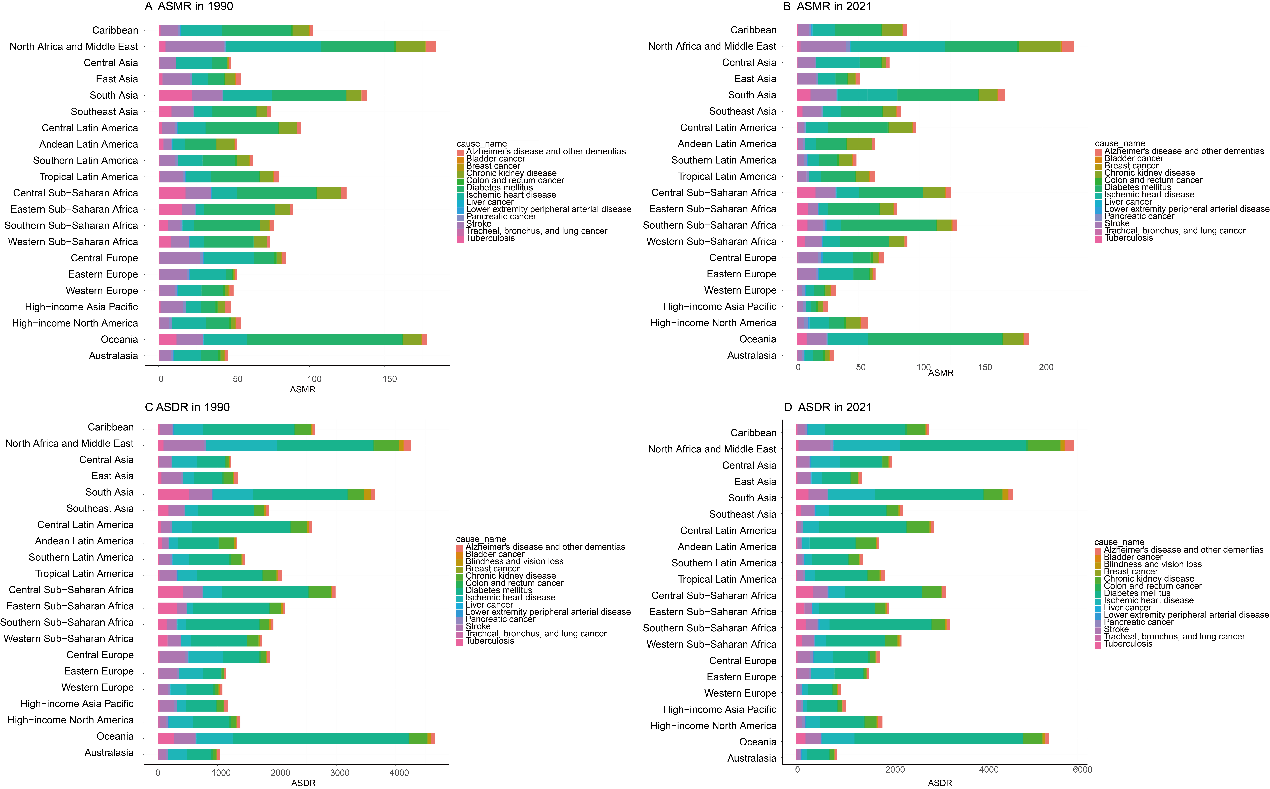


Figure S2 ASMR (A, B) and ASDR (C, D) for disease due to HFPG in 21 GBD regions, 1990 and 2021. ASMR: age - standardized mortality rates. ASDR: age - standardized DALYs rates. DALYs: disability - adjusted life years.


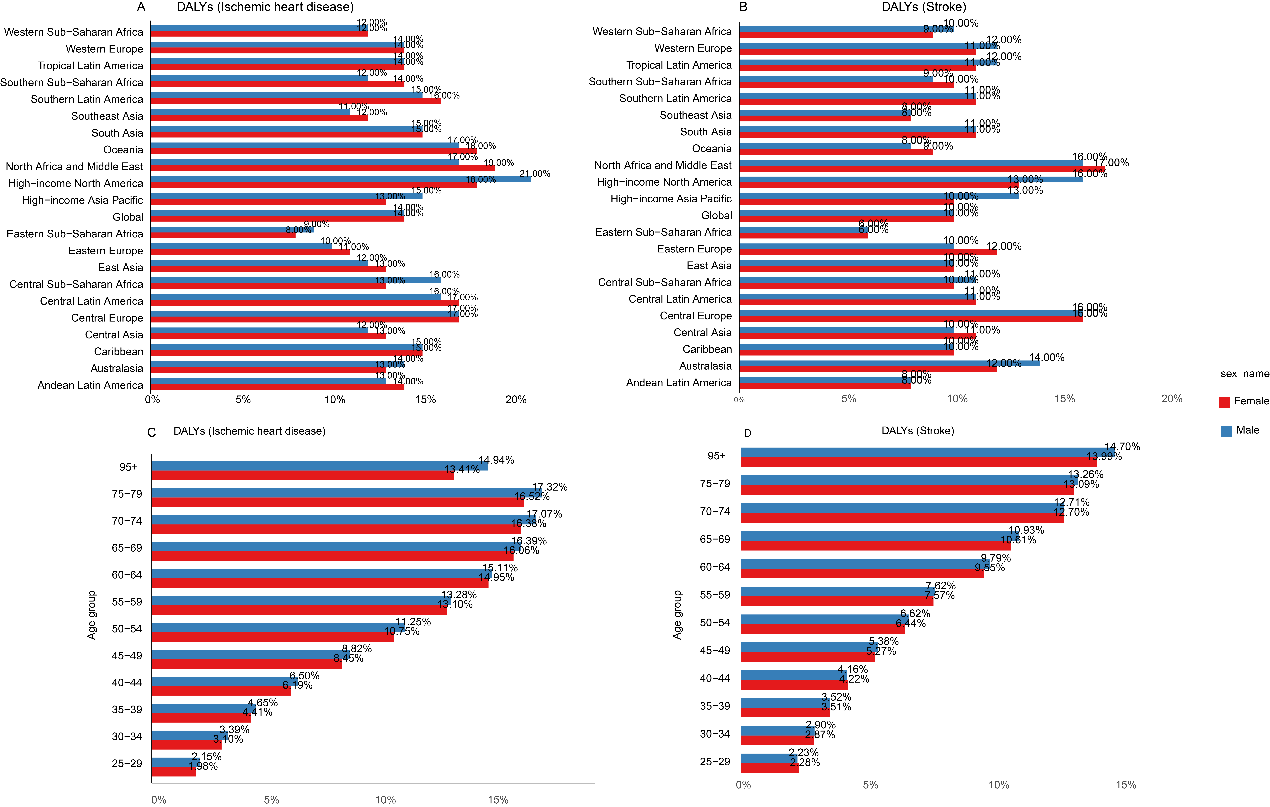


Figure S3 The burden of ischemic heart disease and stroke attributable to HFPG for DALYs are shown. (A) Ischemic heart disease attributable to HFPG in 21 GBD regions. (B) Strok attributable to HFPG 21 GBD regions. (C) Ischemic heart disease attributable to HFPG by age. (D) Strok attributable to HFPG by age.


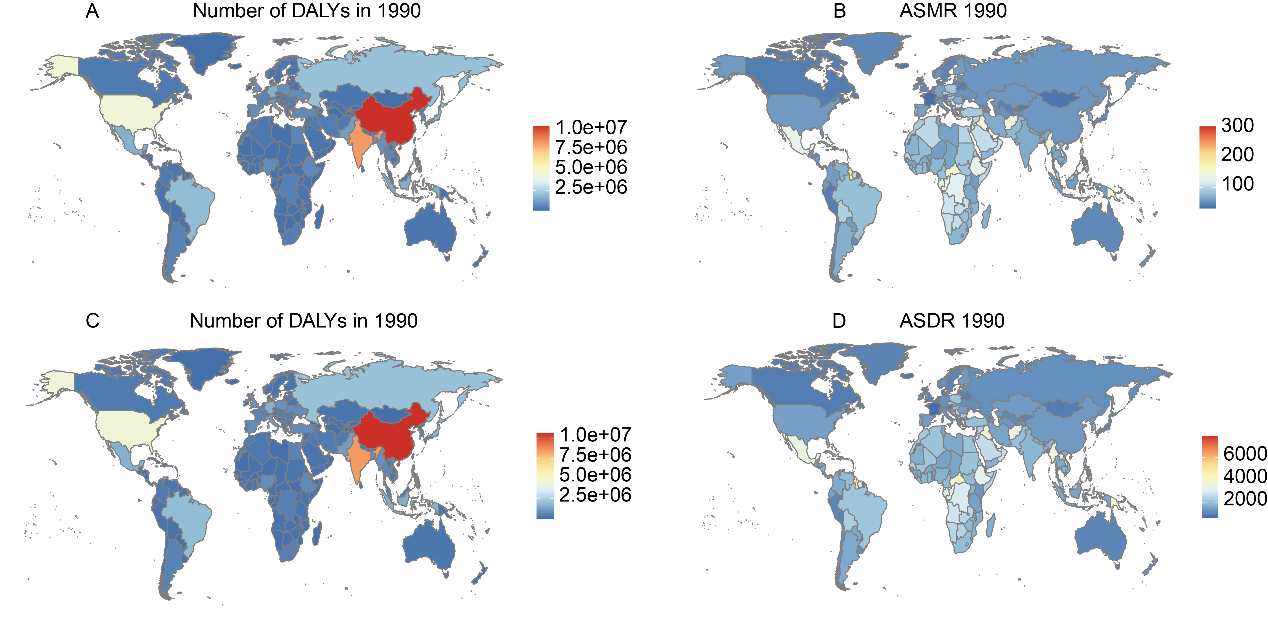


Figure S4 The burden of disease attributable to HFPG for deaths, DALYs cases, mortality, DALYs rates in 1990 across 204 countries and territories. (A) Number of deaths in 1990. (B) ASMR in 1990. (C) Number of DALYs in 1990. (D) ASDR in 1990.
